# Long-term outcomes in COVID-19 patients who recovered from the first wave of the pandemic

**DOI:** 10.1093/nsr/nwac192

**Published:** 2022-09-20

**Authors:** Dan Cui, Simiao Chen, Luzhao Feng, Mengmeng Jia, Yeming Wang, Weijun Xiao, Yanxia Sun, Qiangru Huang, Libing Ma, Zhiwei Leng, Hao Wang, Bin Cao, Weizhong Yang, Juntao Yang, Chen Wang

**Affiliations:** Department of Pulmonary and Critical Care Medicine, The Second Affiliated Hospital of Harbin Medical University, Harbin Medical University, Harbin 150086, China; Department of Pulmonary and Critical Care Medicine, National Center for Respiratory Medicine, Center of Respiratory Medicine, National Clinical Research Center for Respiratory Diseases, China-Japan Friendship Hospital, Beijing 100029, China; Heidelberg Institute of Global Health, Faculty of Medicine and University Hospital, Heidelberg University, Heidelberg 69120, Germany; School of Population Medicine and Public Health, Chinese Academy of Medical Sciences & Peking Union Medical College, Beijing 100005, China; School of Population Medicine and Public Health, Chinese Academy of Medical Sciences & Peking Union Medical College, Beijing 100005, China; School of Population Medicine and Public Health, Chinese Academy of Medical Sciences & Peking Union Medical College, Beijing 100005, China; Department of Pulmonary and Critical Care Medicine, National Center for Respiratory Medicine, Center of Respiratory Medicine, National Clinical Research Center for Respiratory Diseases, China-Japan Friendship Hospital, Beijing 100029, China; School of Population Medicine and Public Health, Chinese Academy of Medical Sciences & Peking Union Medical College, Beijing 100005, China; School of Population Medicine and Public Health, Chinese Academy of Medical Sciences & Peking Union Medical College, Beijing 100005, China; School of Population Medicine and Public Health, Chinese Academy of Medical Sciences & Peking Union Medical College, Beijing 100005, China; School of Population Medicine and Public Health, Chinese Academy of Medical Sciences & Peking Union Medical College, Beijing 100005, China; Department of Respiratory and Critical Care Medicine, the Affiliated Hospital of Guilin Medical University, Guilin, 541001, China; School of Population Medicine and Public Health, Chinese Academy of Medical Sciences & Peking Union Medical College, Beijing 100005, China; School of Population Medicine and Public Health, Chinese Academy of Medical Sciences & Peking Union Medical College, Beijing 100005, China; Department of Pulmonary and Critical Care Medicine, National Center for Respiratory Medicine, Center of Respiratory Medicine, National Clinical Research Center for Respiratory Diseases, China-Japan Friendship Hospital, Beijing 100029, China; Institute of Respiratory Medicine, Chinese Academy of Medical Science, Beijing 100730, China; School of Population Medicine and Public Health, Chinese Academy of Medical Sciences & Peking Union Medical College, Beijing 100005, China; State Key Laboratory of Medical Molecular Biology, Department of Biochemistry and Molecular Biology, Institute of Basic Medical Sciences, Chinese Academy of Medical Sciences & Peking Union Medical College, Beijing 100005, China; Department of Pulmonary and Critical Care Medicine, The Second Affiliated Hospital of Harbin Medical University, Harbin Medical University, Harbin 150086, China; Department of Pulmonary and Critical Care Medicine, National Center for Respiratory Medicine, Center of Respiratory Medicine, National Clinical Research Center for Respiratory Diseases, China-Japan Friendship Hospital, Beijing 100029, China; School of Population Medicine and Public Health, Chinese Academy of Medical Sciences & Peking Union Medical College, Beijing 100005, China; Institute of Respiratory Medicine, Chinese Academy of Medical Science, Beijing 100730, China

**Keywords:** SARS-CoV-2, post-COVID-19 syndrome, follow-up studies

## Abstract

This cross-sectional study evaluated the long-term health effects of coronavirus disease 2019 (COVID-19) in Jianghan District (Wuhan, China). The results showed that 61.4% of COVID-19 patients reported at least one symptom and 8.8% had depressive symptoms at the 17-month follow-up. The proportion of patients with chest radiographic abnormalities in Fangcang shelter hospitals and designated COVID-19 hospitals was 31.6% and 41.1%, respectively, and the proportion of patients with impaired pulmonary diffusion capacity in these hospitals was 52.8% and 60.9%, respectively. Female sex (odds ratio [OR] = 1.48, 95% confidence interval [CI]: 1.16–1.88), severe disease (OR = 1.46, 95% CI: 1.01–2.10) and a higher number of initial symptoms (OR = 1.31, 95% CI: 1.23–1.40) were associated with the development of sequelae symptoms at 17 months. This study involving community-dwelling COVID-19 adults may help determine the long-term effects of COVID-19 during the first pandemic wave. Nonetheless, larger follow-up studies are needed to characterize the post-COVID-19 condition.

## Teaser

This is the first study to characterize post-COVID-19 syndrome and identify risk factors for clinical and psychological sequelae of COVID-19 in two distinct cohorts of patients in China.

## INTRODUCTION

As of 27 July 2022, 570 million cases and >6.4 million COVID-19 deaths were reported worldwide [1] and the time course of recovery has received increased attention. Several post-COVID-19 symptoms and sequelae have been recognized by medical professionals and the World Health Organization officially defined them as post-COVID-19 condition (PCC) in October 2021 [2]. The pulmonary and extrapulmonary clinical manifestations and psychological impact of COVID-19 are a global challenge [3–5].

PCC may persist for up to 12 months after disease onset. A previous cohort study found that 49% of hospitalized COVID-19 patients had at least one sequelae symptom and 26% had anxiety or depression at the 12-month follow-up [3]. In addition, a report from Germany showed that only 23% of recovered COVID-19 patients were completely free of symptoms at the 12-month follow-up and the most frequent symptoms were reduced exercise capacity (56.3%), fatigue (53.1%) and dyspnea (37.5%) [6]. A large national health insurance-based cohort study in the USA reported that the risk of sequelae in older patients, including fatigue, respiratory failure and dementia, after severe acute respiratory syndrome coronavirus 2 (SARS-CoV-2) infection was 11% higher than in the 2020 controls [7].

However, PCC is incompletely understood. It has been reported that 82% of COVID-19 patients in Wuhan, China, were asymptomatic [8] and 81% of symptomatic patients had mild symptoms [9]. In response to the shortage of medical resources during the pandemic, Fangcang shelter hospitals were created and used for the first time in China to isolate and care for non-severe COVID-19 patients [10]. However, the assessment of the health impacts of COVID-19 was based primarily on the follow-up of severely ill patients and lacked appropriate control groups [3,6,11,12]. Furthermore, the evaluation of non-hospitalized patients is based primarily on health-registry data, which have a limited ability to diagnose post-COVID-19 symptoms [13–15] and the prevalence of these symptoms varies widely across studies and patient groups.

This study evaluated the long-term health effects of COVID-19 in patients admitted to Fangcang shelter hospitals and designated hospitals during the first wave of the COVID-19 pandemic and assessed the association between demographic and clinical factors and long-term health effects.

## RESULTS

### Characteristics of the participants

**Figure 1.**
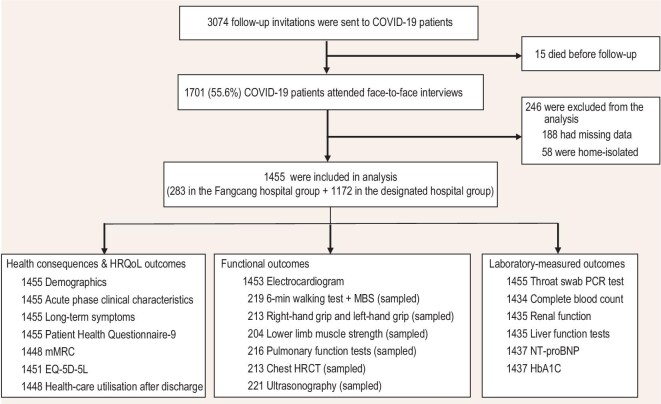
Flow chart of participants. HRQoL, health-related quality of life; mMRC, modified Medical Research Council; EQ-5D-5L, EuroQol five-dimension five-level questionnaire; MBS, modified Borg scale; HRCT, high-resolution computed tomography; NT-proBNP, N-terminal pro-B-type natriuretic peptide; HbA1C, glycated hemoglobin A1c.

This study followed up COVID-19 patients in Jianghan District (Wuhan, China) from 10 June to 25 July 2021. These patients were infected with the original SARS-CoV-2 strain and were diagnosed between December 2019 and April 2020. According to the electronic medical records of the Health Bureau of Jianghan District, 15 patients died after hospital discharge (Supplementary Table S1). None of our patients was reinfected with SARS-CoV-2. A total of 3059 patients with laboratory-confirmed COVID-19 were eligible for follow-up and 1701 (55.6%) participated in face-to-face interviews at a tertiary hospital (Fig. 1). In total, 1455 survivors were included in the analysis; of these, 283 (19.5%) were admitted to 14 Fangcang shelter hospitals and 1172 (80.5%) were admitted to 41 designated COVID-19 hospitals (Supplementary Table S2). The basic configuration of Fangcang shelter hospitals and of a representative designated hospital is shown in Supplementary Table S3.

The mean age of the study population was 58.3 years (standard deviation [SD]: 12.3) and 58.1% (846) of the patients were women (Table 1). Overall, 826 patients (56.8%) had at least one pre-existing comorbidity, especially cardiovascular and cerebrovascular diseases (518 patients [35.6%]) and diabetes (229 patients [15.7%]). Seventy-three participants were asymptomatic (5.0%), 1032 had mild symptoms (70.9%), 144 had moderate symptoms (9.9%) and 206 had severe disease (14.2%). Furthermore, 62% of patients received the SARS-CoV-2-inactivated vaccine after infection. The mean follow-up period was 17 months (SD: 0.6) after symptom onset.

**Table 1.** Characteristics of COVID-19 patients.

	Total cohort (*n* = 1455)	Fangcang shelter hospital group (*n* = 283)	Designated hospital group (*n* = 1172)
**Age**			
**Mean age (SD), years**	58.3 (12.3)	52.8 (10.4)	59.7 (12.3)
18**–**39	127 (8.7%)	38 (13.4%)	89 (7.6%)
40**–**59	578 (39.7%)	157 (55.5%)	421 (35.9%)
≥60	750 (51.6%)	88 (31.1%)	662 (56.5%)
**Gender**			
Male	609 (41.9%)	111 (39.2%)	498 (42.5%)
Female	846 (58.1%)	172 (60.8%)	674 (57.5%)
**Marital status**			
Married	1221 (83.9%)	239 (84.5%)	982 (83.8%)
Non-married	234 (16.1%)	44 (5.7%)	190 (2.6%)
**Education**			
Middle or lower	1039 (71.4%)	190 (67.1%)	849 (72.4%)
College or higher	416 (28.6%)	93 (32.9%)	323 (27.6%)
**Smoking**			
Yes	188 (12.9%)	41 (14.5%)	147 (12.5%)
No	1267 (87.1%)	242 (85.5%)	1025 (87.5%)
**Family income (thousand RMB per year)**			
<60	879 (60.4%)	160 (56.5%)	719 (61.3%)
≥60	576 (39.6%)	123 (43.5%)	453 (38.7%)
**Comorbidities**	826 (56.8%)	119 (42.0%)	707 (60.3%)
Bronchial diseases	61 (4.2%)	6 (2.1%)	55 (4.7%)
Cancers	25 (1.7%)	7 (2.5%)	18 (1.5%)
Cardiovascular and cerebrovascular diseases	518 (35.6%)	64 (22.6%)	454 (38.7%)
Chronic kidney diseases	30 (2.1%)	5 (1.8%)	25 (2.1%)
Diabetes	229 (15.7%)	27 (9.5%)	202 (17.2%)
Digestive diseases	113 (7.8%)	23 (8.1%)	90 (7.7%)
Other diseases	210 (14.4%)	34 (12%)	176 (15.0%)
**Illness severity**			
Asymptomatic	73 (5.0%)	26 (9.2%)	47 (4%)
Mild	1032 (70.9%)	237 (83.7%)	795 (67.8%)
Moderate	144 (9.9%)	11 (3.9%)	133 (11.3%)
Severe or critical	206 (14.2%)	9 (3.2%)^a^	197 (16.8%)
**Acute phase symptoms**			
Fever	989 (68.0%)	177 (62.5%)	812 (69.3%)
Cough	702 (48.2%)	138 (48.8%)	564 (48.1%)
Dyspnea	451 (31.0%)	48 (17.0%)	403 (34.4%)
Fatigue	692 (47.6%)	109 (38.5%)	583 (49.7%)
Nausea or vomiting	158 (10.9%)	31 (11.0%)	127 (10.8%)
Diarrhea	334 (23.0%)	70 (24.7%)	264 (22.5%)
Myalgia	292 (20.1%)	52 (18.4%)	240 (20.5%)
Smell disorders	158 (10.9%)	22 (7.8%)	136 (11.6%)
Taste disorders	267 (18.4%)	46 (16.3%)	221 (18.9%)
**Time from symptom onset to follow-up, months**	16.9 (1.3)	17.0 (1.3)	16.9 (1.2)
**COVID-19 vaccination** ^b^	902 (62.0%)	186 (65.7%)	716 (61.1%)

Data are numbers (%) or means (SD). ^a^All nine severe patients were infected at the early stage of the epidemic and were home-isolated because of the limited supply of medical resources, including hospital beds. These patients moved to Fangcang shelter hospitals starting on February 2020. ^b^Since April 2021, the Chinese government has encouraged those who have recovered from COVID-19 to take SARS-CoV-2 vaccines; therefore, all those who recovered in this study received SARS-CoV-2 vaccines after their infection.

Data on community-dwelling adults without SARS-CoV-2 infection were used as controls. Briefly, 3383 non-infected individuals were recruited from two districts of Wuhan between December 2020 and January 2021. This group answered the same questionnaires as COVID-19 patients to compare the health status of the case and control groups. In total, 1455 matched (1:1) control subjects were included in the final analysis. The mean age of this group was 57.7 years (SD: 11.8) and 58.8% (855) were women. The most common comorbidities were cardiovascular and cerebrovascular diseases (519 patients, 35.7%), diabetes (216 patients, 14.8%) and pulmonary diseases (57 patients, 3.9%).

### Self-reported health effects and health-related quality of life (HRQoL)

**Figure 2.**
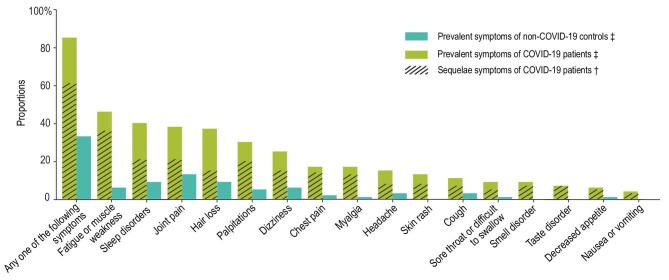
Prevalent symptoms in COVID-19 patients and non-infected controls and sequelae symptoms in COVID-19 patients. ^‡^Prevalent symptoms were defined as existing symptoms at follow-up. ^†^Sequelae symptoms were defined as new and persistent symptoms or symptoms that worsened after COVID-19 and could not be explained by other illnesses.

We list 16 symptoms (including fatigue or muscle weakness, sleep disorders, hair loss, smell disorder, etc.) for participants to choose from (any other if yes, specify additionally). As shown in Fig. 2, the proportion of individuals with at least one prevalent symptom was significantly higher in the case group than in controls (85.1% vs. 32.7%, *P *< 0.001). For each prevalent symptom, the case group reported significantly higher proportions (all *P *< 0.001) than in controls. The percentage of COVID-19 patients with at least one sequelae symptom in the total cohort and in the groups admitted to Fangcang or designated hospitals was 61.4%, 58.3% and 62.2%, respectively. The most frequent sequelae symptoms were fatigue/muscle weakness (35.9%), sleep disorders (20.8%) and joint pain (20.5%) (Supplementary Table S4).

A total of 1451 COVID-19 patients answered the EQ-5D-5L questionnaire. The most common health problems were pain/discomfort (36.5%) and anxiety/depression (27.9%) (Supplementary Table S5). The mean score on the EuroQol Visual Analogue Scale (EQ-VAS) for patients admitted to Fangcang and designated hospitals was 77.8 (SD: 12.9) and 75.2 (SD: 13.4), respectively. On the modified Medical Research Council (mMRC) dyspnea scale, 45.1% of patients had mMRC scores of ≥1 and the percentage of patients with scores of ≥1 in Fangcang and designated hospitals was 39.4% and 46.5%, respectively. In addition, most patients (92.2%) reported being healthy and 23.5% reported being fully healthy.

The analysis of the Patient Health Questionnaire-9 (PHQ-9) showed that 71.9% of patients had no or minimal depressive symptoms and 8.8% had moderate or severe depressive symptoms (score of ≥10). The proportion of patients with moderate or severe depressive symptoms in Fangcang and designated hospitals was 7.4% and 9.1%, respectively. With respect to healthcare utilization after discharge, one-third of COVID-19 patients had visited an outpatient clinic, 20% were admitted to a hospital and 10% had visited an emergency department after discharge (Table 2).

**Table 2.** Health-related quality of life, psychological symptoms and healthcare utilization of COVID-19 patients.

	Total cohort (*n* = 1455)	Fangcang shelter hospital group (*n* = 283)	Designated hospital group (*n* = 1172)
**Health-related quality of life**			
** EQ-5D-5L questionnaire** ^a^			
Mobility	101/1451 (7.0%)	8 (2.8%)	93/1168 (8.0%)
Personal care	16/1451 (1.1%)	0 (0%)	16/1168 (1.4%)
Usual activity	34/1451 (2.3%)	0 (0%)	34/1168 (2.9%)
Pain or discomfort	530/1451 (36.5%)	92 (32.5%)	438/1168 (37.5%)
Anxiety or depression	405/1451 (27.9%)	76 (26.9%)	329/1168 (28.2%)
** EQ-VAS** ^b^	75.7 (13.4)	77.8 (12.9)	75.2 (13.4)
** mMRC score of ≥1**	653/1448 (45.1%)	111/282 (39.4%)	542/1166 (46.5%)
** Perceived current health status**			
Fully healthy	339 (23.3%)	96 (33.9%)	243 (20.7%)
Healthy	1003 (68.9%)	174 (61.5%)	829 (70.7%)
Not healthy, but able to perform self-care	113 (7.8%)	13 (4.6%)	100 (8.5%)
Unable to perform self-care	0 (0%)	0 (0%)	0 (0%)
**Psychological symptoms**			
** PHQ-9 questionnaire scores in the last 2 weeks**			
Mean score (SD)	3.4 (4.0)	3.2 (4)	3.5 (4.0)
None–minimal (0–4)	1046 (71.9%)	212 (74.9%)	834 (71.2%)
Mild (5–9)	281 (19.3%)	50 (17.7%)	231 (19.7%)
Moderate–severe (≥10)	128 (8.8%)	21 (7.4%)	107 (9.1%)
** Healthcare utilization**			
Outpatient clinics	450/1447 (31.1%)	86/280 (30.7%)	364/1167 (31.2%)
Hospitals	293/1448 (20.2%)	44/280 (15.7%)	249/1168 (21.3%)
Emergency departments	142/1448 (9.8%)	29/280 (10.4%)	113/1168 (9.7%)

Data are numbers (%) and means (SD). EQ-5D-5L, EuroQol five-dimension five-level questionnaire; mMRC, modified Medical Research Council; PHQ-9, Patient Health Questionnaire-9. ^a^Detailed results of the EQ-5D-5L questionnaire among COVID-19 patients are shown in Supplementary Table S5. ^b^Quality of life was assessed using the EuroQol Visual Analogue Scale, ranging from 0 (poor health) to 100 (excellent health).

### Pulmonary performance and exercise capacity

Lung-function tests were performed on 216 patients (38 in the Fangcang group and 178 in the designated hospital group). Of these, 125 (59.5%) had impaired pulmonary diffusion (diffusing capacity for carbon monoxide <80% of the predicted value). The percentage of patients with impaired pulmonary diffusion in these two groups was 52.8% and 60.9%, respectively. The percentage of participants with restrictive ventilatory dysfunction (total lung capacity <80% of the predicted value) in the total cohort and in these two groups was 54.6%, 42.1% and 57.3%, respectively.

A total of 213 patients underwent chest high-resolution computed tomography (HRCT) (38 in the Fangcang group and 175 in the designated hospital group). Of these, 84 (39.4%) had at least one radiographic abnormality. The most frequent abnormalities were irregular pleural lines (32.9%), ground-glass opacity (21.1%) and interlobular septal thickening (12.1%). The analysis stratified by the type of hospital yielded similar results.

A total of 219 patients completed the 6-min walk test (6MWT) and the modified Borg dyspnea scale (MBS) (40 patients in the Fangcang group and 179 in the designated hospital group). The average 6MWT distance in these two groups was 547.0 m (SD: 80.0) and 503.3 m (SD: 78.3), respectively. The percentage of individuals with a median 6MWT score below the lower limit of the normal range was 8.2%. Muscle strength was assessed in 213 patients. Of these, 42 (19.0%) had low left-hand grip strength and 28 (12.7%) had low right-hand grip strength (Table 3).

**Table 3.** Pulmonary performances and exercise capacity of COVID-19 patients at 17-month follow-up.

	Total cohort	Fangcang shelter hospital group	Designated hospital group
**Lung function**			
Number of patients	216	38	178
FEV_1_ < 80%, % of predicted	17 (8.1%)	0 (0%)	17 (9.8%)
FVC < 80%, % of predicted	21 (9.7%)	1 (2.6%)	20 (11.2%)
FEV_1_/FVC < 70%	5 (2.3%)	0 (0%)	5 (2.8%)
TLC < 80%, % of predicted	118 (54.6%)	16 (42.1%)	102 (57.3%)
FRC < 80%, % of predicted	103 (49.0%)	18 (50.0%)	85 (48.9%)
DLCO < 80%, % of predicted^a^	125 (59.5%)	19 (52.8%)	106 (60.9%)
DLCO/VA < 80%, % of predicted^a^	45 (20.8%)	8 (21.1%)	37 (20.8%)
**High-resolution chest tomography**			
Number of patients	213	38	175
At least one radiographic abnormality	84 (39.4%)	12 (31.6%)	72 (41.1%)
Irregular pleural lines	70 (32.9%)	10 (26.3%)	60 (34.3%)
Ground-glass opacities	45 (21.1%)	4 (10.5%)	41 (23.4%)
Subpleural line	21 (9.9%)	4 (10.5%)	17 (9.7%)
Interlobular septal thickening	27 (12.7%)	1 (2.6%)	26 (14.9%)
Reticular pattern	8 (3.8%)	0 (0%)	8 (4.6%)
Consolidation	0 (0%)	0 (0%)	0 (0%)
**6MWT**			
Number of patients	219	40	179
Distance walked in 6 min, m	511.3 (78.7)	547.0 (80.0)	503.3 (78.3)
Less than the lower limit of the normal range^b^	18/219 (8.2%)	2/40 (5.0%)	16/179 (8.9%)
Maximal MBS during 6MWT	1.6 (0.8)	1.5 (0.7)	1.6 (0.8)
**Muscle strength**			
Number of patients	213	39	174
Low LHG strength^c^	42/221 (19.0%)	8/40 (20.0%)	34/181 (18.8%)
Low RHG strength^c^	28/221 (12.7%)	2/40 (5.0%)	26/181 (14.4%)
Lower-limb muscle strength (LMS), s	34.9 (20.4)	41.6 (25.5)	33.4 (18.8)

Data are numbers (%) or means (SD). FEV_1_, forced expiratory volume in 1 s; FVC, forced vital capacity; TLC, total lung capacity; FRC, functional residual capacity; DLCO, diffusion capacity of carbon monoxide; VA, alveolar volume; 6MWT, 6-min walk test; MBS, modified Borg dyspnea scale; LHG, left-hand grip; RHG, right-hand grip; LMS, lower-limb muscle strength. ^a^Carbon monoxide diffusion capacity was not corrected for hemoglobin. ^b^Predicted values were calculated according to the method described by Enright and Sherrill. The lower limit of the normal range was calculated by subtracting 153 m from the predicted value for men and 139 m for women. ^c^Low muscle strength was defined as handgrip strength of <26 kg for men and <18 kg for women.

### Extrapulmonary sequelae

The results of laboratory tests are shown in Supplementary Table S6. In the case group, 13.6% had glycated hemoglobin A1c (HbA1c) levels of ≥6.5%, 11.5% had an estimated glomerular filtration rate of <60 mL/min and 7.5% had a leukocyte count of <4.0 × 10^9^ per liter at the 17-month follow-up. As expected, COVID-19 patients who received two doses of SARS-CoV-2-inactivated vaccine after infection had higher IgG antibody titers than those who received one dose and those who were unvaccinated. In addition, all patients had negative nucleic acid test results at follow-up.

### Risk factors for sequelae symptoms, depressive symptoms, radiographic abnormalities and impaired pulmonary diffusion

Risk factors for sequelae symptoms, depressive symptoms, radiographic abnormalities and impaired pulmonary diffusion were assessed using multivariable logistic regression models adjusted for age, sex, cigarette smoking, pre-existing comorbidities, disease severity, number of initial symptoms, public health interventions and SARS-CoV-2 vaccination status.

**Figure 3.**
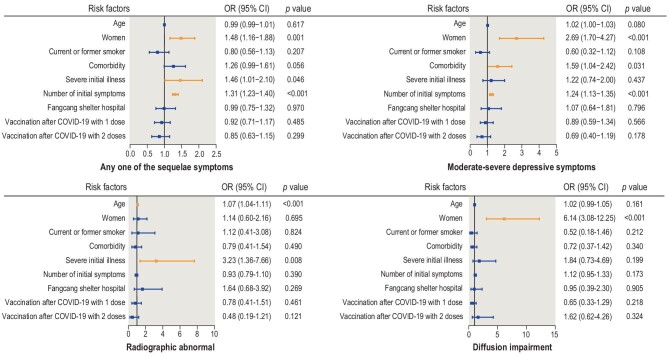
Risk factors for sequelae symptoms, depressive symptoms, radiographic abnormalities and impaired pulmonary diffusion in COVID-19 patients.

The results of the multivariable logistic regression analysis indicated that females had a higher risk of sequelae symptoms (OR: 1.48, 95% CI: 1.16–1.88), depressive symptoms (OR: 2.69, 95% CI: 1.70–4.27) and impaired pulmonary diffusion (OR: 6.14, 95% CI: 3.08–12.25) than males at the 17-month follow-up. There were no significant sex differences in radiographic abnormalities. Age was positively associated with radiographic abnormalities, with a 7% increase in risk (OR: 1.07, 95% CI: 1.04–1.11) for each year of age. Compared with non-severe patients, severe patients had an OR of 1.46 (95% CI: 1.01–2.10) for sequelae symptoms and 3.23 (95% CI: 1.36–7.66) for radiographic abnormalities. The number of initial symptoms was associated with sequelae symptoms (OR: 1.31, 95% CI: 1.23–1.40) and depressive symptoms (OR: 1.24, 95% CI: 1.13–1.35). Pre-existing comorbidities were associated with an increased risk of depressive symptoms (OR: 1.24, 95% CI: 1.13–1.35). There was no significant association between public health interventions or SARS-CoV-2 vaccination status and the four outcomes mentioned above (Fig. 3).

The results of *post hoc* subgroup analysis according to follow-up period (<17, 17 and >17 months) indicated that the number of initial symptoms and female sex were risk factors for sequelae symptoms (Supplementary Table S7). The ORs for the number of initial symptoms decreased throughout follow-up, with an OR of 1.40 (95% CI: 1.22–1.60), 1.31 (95% CI: 1.21–1.42) and 1.17 (95% CI: 0.98–1.39) for the follow-up of <17, 17 and >17 months, respectively. Conversely, the ORs for the female sex increased throughout follow-up, with an OR of 1.07 (95% CI: 0.66–1.73), 1.50 (95% CI: 1.11–2.04) and 2.40 (95% CI: 1.21–4.77), respectively.

## DISCUSSION

We assessed the long-term health effects of COVID-19 in community-dwelling adult COVID-19 survivors in Jianghan District—the first urban area to report COVID-19 cases in China—17 months after disease onset. The rates of prevalent symptoms were remarkably higher in COVID-19 patients than in matched non-infected controls. Many patients had physical or psychological symptoms, displayed pulmonary radiographic abnormalities or had recovered slowly in terms of diffusion impairment after 17 months regardless of whether they had been cared for in Fangcang shelter hospitals or designated hospitals. Moreover, female sex, number of initial symptoms and disease severity were associated with sequelae symptoms at 17 months.

Studies have shown that non-severe and severe cases of COVID-19 are associated with PCC [5,13,14,16]. Consistently with our previous findings in hospitalized patients [3,17], two-thirds of infected patients had at least one sequelae symptom at 17 months and fatigue/muscle weakness was the most persistent condition. The UK Office for National Statistics reported that 3% of the British population had ongoing symptoms following COVID-19 and 56% of this group had fatigue [18]. Long-term health effects also occur in other viral diseases, including Ebola, dengue and SARS [19]. Possible explanations may include (i) a hidden reservoir of viruses that drives chronic inflammation; (ii) aberrant immune responses, persistent immune activation and autoimmunity; (iii) dysbiosis of the microbiome or virome; and (iv) limited ability to repair damaged lung tissue [20–22].

Our analysis indicated that female sex, number of initial symptoms and disease severity were associated with sequelae symptoms, consistently with previous studies [13,15,16]. Additionally, the effects of the number of initial symptoms decreased while the effects of demographic factors increased as the disease progressed. However, the long-term health effects were not significantly associated with public health interventions and SARS-CoV-2 vaccination status after COVID-19. A longer follow-up is required to elucidate PCC and clinical recovery.

PCC also impairs the HRQoL and mental health. An investigation in Wuhan indicated that 34.72% and 28.47% of patients with COVID-19 had symptoms of anxiety and depression during the acute phase, respectively [23]. In our cohort, one-third of patients had pain/discomfort or anxiety/depression and 10% had depressive symptoms 17 months after onset. Consistently with this observation, one-third of SARS and Middle East Respiratory Syndrome (MERS) patients experienced anxiety or depression 6 months after discharge and their quality of life was poorer at 12 months after discharge [24]. The impairment of HRQoL and mental health may be due to uncertain treatment, social isolation and stigma [25,26].

Studies of viral infections associated with pulmonary involvement, including SARS, MERS and H1N1 influenza, suggest that functional impairment and radiological abnormalities persist after hospital discharge [27]. In line with this result, we found that radiographic abnormalities and impaired lung diffusion persisted for ≤17 months after discharge in most COVID-19 patients. Previous studies have shown that ground-glass opacity was the most common radiographic abnormality at 3-, 6- and 12-month follow-up [3,28,29]. In contrast, irregular pleural lines were the most common type of abnormality in our population and no consolidations were observed at 17-month follow-up, indicating that radiographic abnormalities improved, albeit slowly. Additionally, a typical radiographic pattern associated with impaired pulmonary diffusion and decreased total lung capacity was common, suggesting that pulmonary injury was due to parenchymal infiltration, lung epithelial damage and interstitial/pulmonary vascular abnormalities [30–32].

To our knowledge, this is the first cross-sectional study to evaluate community-dwelling adult COVID-19 patients with asymptomatic or severe disease in China. Other unique characteristics of our study were cohort composition (20% were isolated in Fangcang shelter hospitals and the remaining patients came from >40 designated hospitals), the assessment of long-term health outcomes in patients admitted to Fangcang shelter hospitals, the inclusion of non-infected controls in the analysis and the use of standardized study procedures building off our previous follow-up studies.

Notwithstanding, this study has limitations. First, there was only one follow-up visit; thus, additional follow-up studies are needed to assess PCC. Second, the effects of SARS-CoV-2 vaccination, SARS-CoV-2 variants and reinfection were not evaluated. Third, the retrospective analysis of acute phase data may lead to recall bias. In this respect, strict quality control of questionnaires can reduce bias. Fourth, the conclusions cannot be generalized to other populations because the study recruited patients living in one Chinese district and 55.6% of eligible COVID-19 survivors voluntarily answered the questionnaire, potentially causing selection and survivor bias. However, all patients were invited to answer the questionnaire and were recruited from 14 Fangcang shelter hospitals and 41 designated hospitals, which increased representativeness to some degree.

## CONCLUSIONS

The prevalence of symptoms and sequelae was remarkable in COVID-19 patients, regardless of whether these patients were treated in Fangcang shelter hospitals or designated hospitals. Further studies are needed to characterize clinical recovery and PCC's pathophysiology, and improve disease management.

## METHODS

### Study design

This community-based cross-sectional study was conducted between 10 June and 25 July 2021. Individuals aged ≥18 years living in Jianghan District (Wuhan, China) and those with a positive result on a nasopharyngeal/oropharyngeal swab reverse transcriptase-polymerase chain reaction test for SARS-CoV-2 (original strain) were eligible. The exclusion criteria were pregnant or breastfeeding women, patients with dementia or psychological disorders, patients with missing baseline data (sex and disease severity) and home-isolated COVID-19 patients. The flow diagram is shown in Fig. 1.

Community-dwelling adults without SARS-CoV-2 infection were used as non-infected controls [3]. The recruitment and propensity score matching methods for controls are shown in Appendix 2. Self-reported symptoms in cases and controls were compared to determine the health impact of COVID-19.

The study was approved by the Ethics Review Board of the Institute of Pathogen Biology, Chinese Academy of Medical Sciences (IPB-2020–22) and the Research Ethics Committee of the hospital (2021001, 20210208). The participants were informed about the study protocols and gave written informed consent.

### Procedures

Patient information was obtained from the electronic medical records of the Health Bureau of Jianghan District. Appointments for follow-up visits were scheduled by the staff of the Health Bureau via telephone. All patients were contacted in order of the communities in which they lived.

Follow-up was performed at the physical examination center of the tertiary hospital. All patients were interviewed face to face by trained physicians and data on demographic characteristics, health status and initial symptoms were collected using a questionnaire.

For follow-up assessment, long-term outcomes in COVID-19 patients were defined as a collection of outcomes, including physiological or clinical outcomes, life impact outcomes and survival. The patients were asked to report their prevalent and sequelae symptoms, HRQoL scores (EQ-5D-5L questionnaire, EQ-VAS), psychological status (PHQ-9), vaccination status and healthcare utilization after discharge. Prevalent symptoms were defined as existing symptoms at follow-up. Sequelae symptoms were defined as new and persistent symptoms or symptoms that worsened after SARS-CoV-2 infection and could not be explained by other illnesses [33]. Venous blood and throat swab samples were collected from all patients at follow-up. Patients with mild, moderate and severe COVID-19 (4:3:3 ratio) were selected by random sampling and were invited to undergo additional examinations including a 6MWT, muscle strength tests, pulmonary function tests and chest HRCT examinations. The outcome measures and assessment tools are described in Appendices 4–6.

The diagnosis and definition of disease severity were based on the Diagnosis and Treatment Scheme for COVID-19 released by the National Health Commission of China [34]. For convenience of description, asymptomatic, mild and moderate cases were regarded as non-severe disease and severe and critical cases were regarded as severe disease. We considered each patient's most severe status as the final illness severity; therefore, all patients treated in a designated hospital were considered to be in the designated hospital group.

### Statistical analysis

Continuous variables were expressed as the mean ± SD or median (interquartile range [IQR]) and categorical variables were expressed as numbers and percentages. Demographic and clinical data were collected by the hospital staff. Persistent symptoms in COVID-19 patients and non-infected controls were compared using the χ² test.

The effect of sequelae symptoms, depressive symptoms, radiographic abnormalities and impaired pulmonary diffusion was evaluated using multivariate logistic regression models adjusted for potential confounders, including age, smoking status and comorbidities. In addition, a *post hoc* subgroup analysis was performed according to the follow-up period (<17, 17 and >17 months) to analyse the association between risk factors for COVID-19 sequelae symptoms.

Statistical analyses were performed using SAS software version 9.4 (SAS Institute Inc., Cary, NC, USA) and GraphPad Prism version 9.1 (GraphPad Software, San Diego, CA, USA).

## DATA AVAILABILITY

The data used in the study are restricted and therefore not publicly available. However, the data are available from the authors upon reasonable request and with the permission of the institution.

## Supplementary Material

nwac192_Supplemental_File
