# Supplementary material for: Long-term outcomes in COVID-19 patients who recovered from the first wave of the pandemic
Source: Natl Sci Rev. 2022 Sep 20;9(11):nwac192. doi: 10.1093/nsr/nwac192 (PMC9718796; doi:10.1093/nsr/nwac192)
Supplement: nwac192_Supplemental_File [file nwac192_supplemental_file.docx]

# Supplementary Materials

**Long-term outcomes in COVID-19 patients recovered from the first wave of the pandemic**

Contents

[Supplementary Materials 1](#_Toc113270079)

[Methods supplement 1](#_Toc113270080)

[a. Definition of illness severity in the acute phase: 1](#_Toc113270081)

[b. Fangcang shelter hospital admission criteria: 1](#_Toc113270082)

[c. Fangcang shelter hospital transfer criteria:^2^ 1](#_Toc113270083)

[d. Fangcang shelter hospital discharge criteria:^2^ 1](#_Toc113270084)

[e. Designated hospital discharge criteria:^3^ 2](#_Toc113270085)

[f. Matched non-COVID controls:^4^ 2](#_Toc113270086)

[g. Study procedures 3](#_Toc113270087)

[h. Outcome measures 4](#_Toc113270088)

[i. Sample size estimated 6](#_Toc113270089)

[Table S1 Characteristics of patients who died after discharge from hospital 8](#_Toc113270090)

[Table S2 List of management sites of COVID-19 patients during acute phase 9](#_Toc113270091)

[Table S3 The basic configuration of Fangcang shelter hospitals and a representative of the designated hospitals 11](#_Toc113270092)

[Table S4 Self-reported symptoms of COVID-19 patients and non-COVID-19 controls 12](#_Toc113270093)

[Table S5 Detailed results of EQ-5D-5L questionnaire of the COVID-19 patients 13](#_Toc113270094)

[Table S6 Extra-pulmonary organ performance of the community-based COVID-19 patients 14](#_Toc113270095)

[Table S7 Post hoc analysis for sequelae symptoms by dividing patients with different follow-up duration 15](#_Toc113270096)

# Methods supplement

## a. Definition of illness severity in the acute phase:

Diagnosis and definition of the severity of the acute phase of illness were based on the Diagnosis and Treatment Scheme for COVID-19 released by the National Health Commission of China.[1] Asymptomatic infection is defined by a positive result for SARS-CoV-2 on a virologic test without development of COVID-19 symptoms. Mild illness is defined by mild clinical symptoms and absence of pneumonia as detected by imaging. Moderate illness is defined by symptoms such as fever and respiratory tract symptoms and the manifestation of pneumonia as detected by imaging. Severe illness is defined by any of the following criteria: dyspnea or respiratory rate ≥30 breaths/min, oxygen saturation ≤93% at a resting state, or arterial partial pressure of oxygen (PaO_2_)/oxygen concentration (FiO_2_) ≤300 mmHg. Patients with >50% lesion progression within 24 to 48 hours in lung imaging should also be treated as severe cases. Critical cases are defined by any of the following criteria: occurrence of respiratory failure requiring mechanical ventilation, presence of shock, or other organ failure that requires monitoring and treatment in an intensive care unit.

b. Fangcang shelter hospital admission criteria:^[2]^

(1) Positive COVID-19 test with mild to moderate illness; (2) Ability to walk and live independently; (3) Absence of severe chronic diseases, including hypertension, diabetes, coronary heart disease, malignancy, structural lung disease, pulmonary heart disease, and immunosuppression; (4) No history of mental health conditions; (5) <65 years old; and (6) Negative influenza test.

## **c. Fangcang shelter hospital transfer criteria:**^[2]^

If patients met any of the following clinical criteria, they were quickly transferred to designated higher-level hospitals: respiration rate of 30 beats per min or higher; blood oxygen saturation of 93% or lower; a partial pressure of arterial oxygen to fraction of inspired oxygen ratio of 300 mm Hg or less; lung imaging showing a greater than 50% progression of lesions within 24–48 h; or the identification or development of severe chronic diseases, including hyper tension, diabetes, coronary heart disease, cancer, structural lung disease, pulmonary heart disease, or immunosuppression.

## **d. Fangcang shelter hospital discharge criteria:**^[2]^

If the patients met all of the following criteria: normal body temperature for >3 days, significant improvement of respiratory symptoms, lung imaging showing obvious absorption of inflammation, and negative nucleic acid tests results for COVID-19 at two consecutive times with a sampling interval of at least 1 day.

## **e. Designated hospital discharge criteria:**^[3]^

No fever for 3 consecutive days, improvement in respiratory symptoms, obvious resolution and recovery of acute lesion in lung imaging, and two negative test results for SARS-CoV-2 24 h apart).

## f. Matched non-COVID controls:[4]

The community-dwelling non-COVID controls were recruited in another study by our team (a follow-up study of hospitalized patients at Jin Yin-tan Hospital) to assess whether the health status of COVID-19 patients had returned to that of the general population. Briefly, the non-COVID controls were recruited from two districts of Wuhan city between Dec 24, 2020, and Jan 16, 2021, by a 4-stage stratified sampling method. All the recruited 3383 non-COVID controls received the same physical exams and questionnaires as the COVID-19 patients. The inclusion and exclusion criteria of non-COVID controls are as follows:

Inclusion criteria:

1. At least 20 years of age

Exclusion criteria:

1. History of laboratory-confirmed SARS-CoV-2 infection

2. Unable to complete the associated questionnaires, such as dementia and psychotic disease

3. Inability to move autonomously

We used PSM to control for confounding between COVID-19 patients and non-COVID controls. The propensity score is the probability of five potential confounders (age, sex, and comorbidity with bronchial diseases, cardiovascular and cerebrovascular diseases, and diabetes) based on a logistic regression model and a caliper width of 0.2. For each COVID-19 patient, we randomly sampled up to one non-COVID control with a matched propensity score, without replacement. Finally, 1455 non-COVID controls matched with COVID-19 patients were included in the analyses.

## g. Study procedures

|  | **Fangcang shelter hospital patients** | **Designated hospital patients** |
| --- | --- | --- |
| Eligibility screening | X | X |
| Stratified sampling procedures | X | X |
| Face-to-face interview | X | X |
| Informed consent | X | X |
| Demographic data | X | X |
| Medical history | X | X |
| Acute phase background | X | X |
| **Physical examination** | X | X |
| **Self-reported health consequences** |  |  |
| Perceived current health status | X | X |
| Questionnaire on prevalent and sequelae symptoms | X^a^ | X^a^ |
| mMRC dyspnoea scale | X | X |
| PHQ-9 | X | X |
| **HRQoL** |  |  |
| EQ-5D-5L Questionnaire | X | X |
| EQ-VAS | X | X |
| Health-care use | X | X |
| **Laboratory tests** |  |  |
| Blood specimen for complete blood count | X | X |
| Blood specimen for renal function* | X | X |
| Blood specimen for liver function† | X | X |
| Blood specimen for HbA1C | X | X |
| Blood specimen for NT-proBNP ‡ | X | X |
| Nucleic throat swab nucleic acid tests | X | X |
| **Exercise capacity** |  | X |
| 6-min walking test (6MWT) | X^b^ | X^b^ |
| Modified Borg Dyspnea Scale (MBS) | X^b^ | X^b^ |
| Right-hand grip & Left-hand grip | X^b^ | X^b^ |
| Lower limb muscle strength | X^b^ | X^b^ |
| **Pulmonary performance** |  |  |
| Pulmonary function tests | X^b^ | X^b^ |
| Lung HRCT | X^b^ | X^b^ |
| **Extrapulmonary performance** |  |  |
| Electrocardiogram (ECG) | X | X |
| Echocardiography | X^b^ | X^b^ |
| Abdominal ultrasound | X^b^ | X^b^ |
| mMRC=modified British Medical Research Council. PHQ-9=Patient Health Questionnaire-9. EQ-5D-5L=EuroQol five-dimension five-level questionnaire. EQ-VAS=EuroQol Visual Analogue Scale. HbA1C= glycated hemoglobin A1C. NT-proBNP=NT-proB-type Natriuretic Peptide. HRCT=high resolution computed tomography.  a: Symptom was further divided into prevalent symptom and sequelae symptom  b: A stratified disproportional random sampling procedure according to illness severity was used to select patients to undergo 6MWT, combined with MBS, muscle strength tests, pulmonary function test, and HRCT.  * Renal function includes creatine, and estimate glomerular filtration rate (eGFR).  † Liver function includes alanine aminotransferase (ALT), aspartate aminotransferase(AST), albumin, total protein, total bilrubin and direct bilrubin. | | |

## h. Outcome measures

**Outcome measures in COVID-19 patients:**

1. Perceived current health status
2. Prevalent and sequelae symptoms
3. mMRC dyspnoea score.
4. PHQ-9.
5. HRQoL, including EQ-5D-5L questionnaire and EQ-VAS.
6. Outpatient visit and hospital admission after discharge.
7. 6MWT and combined with Modified Borg Dyspnea Scale (MBS)
8. Muscle strength tests, including LHG, RHG, and LMS
9. Lung function parameters include forced expiratory volume in one second (FEV_1_), forced vital capacity (FVC), total lung capacity (TLC), functional residual capacity (FRC), and diffusion capacity for carbon monoxide (DLCO).
10. Lung imaging includes abnormal CT, ground glass opacity, irregular lines, subpleural line, interlobular septal thickening, reticular pattern, and consolidation.
11. Laboratory test results include complete blood count, creatine, eGFR, ALT, AST, albumin, total protein, total bilrubin and direct bilrubin, NT-proBNP, HbA1c, and nucleic throat swab nucleic acid tests.

**Outcome assessment tool**

1. The perceived current health status, using a four-point Likert scale composed of “cannot take care of themselves,” “not healthy, but able to take care themselves,” “basically healthy,” and “fully healthy.” The latter two categories were combined to “basically/fully healthy” for the purpose of statistical analysis.
2. The symptom questionnaire for participants was shown in our previous study[4].
3. The mMRC dyspnoea scale[5] to record the level of dyspnoea with physical activity.^2^ Details of this scoring system are: 0, no breathlessness except on strenuous exercise; 1, shortness of breath when hurrying on the level or walking up a slight hill; 2, walks slower than people of the same age on the level because of breathlessness or has to stop to catch breath when walking at their own pace on the level; 3, stops for breath after walking approximately 100 m or after a few minutes on the level; and 4, too breathless to leave the house, or breathless when dressing or undressing.
4. The PHQ-9[6] was marked with four-point responses ranging from “not at all” to “nearly every day” over the past two weeks. Scores were categorized as none/minimal (0-4), mild (5-9), and moderate-severe (≥10), which are the validated clinical cut points.
5. The EQ-5D-5L questionnaire[7] and the EQ-VAS[8] to evaluate the health related quality of life. The EQ-5D-5L is a validated questionnaire to evaluate patient quality of life by assessment of the following five factors: mobility, self-care, usual activities, pain or discomfort, and anxiety or depression. Categorisation within each factor is divided into five levels that range from no problems to extreme problems. The EQ-VAS is a patient’s subjective assessment of generic health ranging from 0 to 100, with higher scores representing better subjective health experience.
6. A questionnaire was used to record the self-reported outpatient visit and hospital admission.
7. 6-min walking test was done according to the ATS practical guidelines.[9] Each follow-up patient walked on the flat ground as fast as possible without oxygen inhalation and completed the 6MWD test independently. Modified Borg Dyspnea Scale (MBS), referred to patients perceived exertion during submaximal exercise (range, 0–10), was asked before and after 6MWT to investigate the exercise capacity.
8. Muscle strength tests: right-hand grip (RHG), left-hand grip (LHG), and lower limb muscle strength (LMS). Handgrip strength was measured using a Baseline hydraulic hand dynamometer (Evaluation Instruments, New York, U.S.A) with the elbow straight in the standing position. The maximal strength values of two trials for each hand were averaged for the analysis. Low muscle strength was defined as a handgrip strength of < 26 kg and < 18 kg in men and women, respectively.[10] LMS was measured with the static squat test.[11] This involved a squat against the wall with both feet flat on the ground approximating a 90° angle at hip and knees. Time in seconds patients could remain in squatting position was recorded.
9. The pulmonary function test was done in the Lung Function Laboratory using the Quark PFT Series Lung Testing Instruments (COSMED, Italia) according to American Thoracic Society guidelines.[12]
10. Chest HRCT was in the supine position during end-inspiration (UNITED IMAGING uCT780 CT scanner). Images were reconstructed at 1.5 mm slice thickness, with 1.5 mm increment, 512 mm × 512 mm. The final chest CT images during the hospital stay and the follow-up image were cross-compared. The CT features were evaluated by two experienced radiologists and one pulmonologist.
11. All laboratory tests were performed in clinical laboratory of the hospital.

## i. Sample size estimated

We tried to invite all recorded COVID-19 survivors, and 1455 were eligible for our study. A sample size of 1455 participants has a power of 97% (calculated using the normal approximation method) to detect a difference of 0.05 in the proportion of responders between the null and the alternative hypothesis, using an exact two-tailed test with a level of significance (alpha) of 0.05.

Reference

1. China., NHCotPsRo. *Interim diagnosis and treatment of 2019 novel coronavirus pneumonia. 7th ed.* <http://www.nhc.gov.cn/yzygj/s7653p/202003/46c9294a7dfe4cef80dc7f5912eb1989.shtml> (accessed 8 Feb 2022)

2. China., TNHCotPsRo. *Manual for working in Fangcang shelter hospitals (3rd edn; in Chinese)*. <https://mp.weixin.qq.com/s/va9vs4HuP8wRQM5fALQcrg> Date accessed: February 08, 2022

3. Commission, CNH. *Chinese clinical guidance for COVID-19 pneumonia diagnosis and treatment*. <http://kjfy.meetingchina.org/msite/news/show/cn/3337.html> Date accessed: February 08, 2022

4. Huang, L, Yao, Q, Gu, X*, et al.* 1-year outcomes in hospital survivors with COVID-19: a longitudinal cohort study. *Lancet (London, England)*. 2021; **398**(10302): 747-58.

5. Mahler, DA, Wells, CK. Evaluation of clinical methods for rating dyspnea. *Chest*. 1988; **93**(3): 580-6.

6. Levis, B, Benedetti, A, Thombs, BD. Accuracy of Patient Health Questionnaire-9 (PHQ-9) for screening to detect major depression: individual participant data meta-analysis. *BMJ (Clinical research ed)*. 2019; **365**: l1476.

7. Herdman, M, Gudex, C, Lloyd, A*, et al.* Development and preliminary testing of the new five-level version of EQ-5D (EQ-5D-5L). *Qual Life Res*. 2011; **20**(10): 1727-36.

8. Rabin, R, de Charro, F. EQ-5D: a measure of health status from the EuroQol Group. *Ann Med*. 2001; **33**(5): 337-43.

9. ATS statement: guidelines for the six-minute walk test. *American journal of respiratory and critical care medicine*. 2002; **166**(1): 111-7.

10. Chen, L-K, Liu, L-K, Woo, J*, et al.* Sarcopenia in Asia: consensus report of the Asian Working Group for Sarcopenia. *J Am Med Dir Assoc*. 2014; **15**(2).

11. Cho, M. The effects of modified wall squat exercises on average adults' deep abdominal muscle thickness and lumbar stability. *J Phys Ther Sci*. 2013; **25**(6): 689-92.

12. Standardization of Spirometry, 1994 Update. American Thoracic Society. *American journal of respiratory and critical care medicine*. 1995; **152**(3): 1107-36.

# Table S1 Characteristics of patients who died after discharge from hospital

| **No.** | **Sex** | **Age, years** | **Illness severity** | **Cause of death** | **Time of death** |
| --- | --- | --- | --- | --- | --- |
| 1 | Woman | 90 | Severe | Cerebral infarction | 2020.4 |
| 2 | Woman | 89 | Severe | Exacerbation of underlying disease | 2020.NA |
| 3 | Man | 87 | Moderate | Exacerbation of chronic obstructive pulmonary disease | 2020.9 |
| 4 | Man | 87 | Moderate | Exacerbation of underlying disease | 2020.NA |
| 5 | Woman | 86 | Mild | Exacerbation of underlying disease | 2020.NA |
| 6 | Man | 79 | Severe | Exacerbation of chronic obstructive pulmonary disease | 2021.1 |
| 7 | Man | 79 | Severe | Exacerbation of underlying disease | 2020.NA |
| 8 | Woman | 79 | Mild | Heart and respiratory failure | 2020.NA |
| 9 | Man | 78 | Severe | Lung infection and coronary artery disease | 2021.7 |
| 10 | Woman | 78 | Mild | Exacerbation of underlying disease | 2020.9 |
| 11 | Man | 69 | Mild | Exacerbation of underlying disease | 2020.NA |
| 12 | Woman | 63 | Mild | Myocardial infarction | 2021.2 |
| 13 | Man | 59 | Mild | Exacerbation of underlying disease | 2020.NA |
| 14 | Man | 56 | Moderate | Exacerbation of underlying disease | 2020.NA |
| 15 | Man | 50 | Moderate | Renal failure | 2021.6 |

# Table S2 List of management sites of COVID-19 patients during acute phase

| **No.** | **Designated hospital** | **No. of participants** | **Hospital tier**‡ |
| --- | --- | --- | --- |
| 1 | Union Hospital Tongji medical college Huazhong university of science and technology | 155 | 3 |
| 2 | Hubei Provincial Hospital of Integrated Chinese and Western Medicine | 129 | 3 |
| 3 | Cancer Center of Union Hospital | 127 | 3 |
| 4 | Huoshenshan Hospital | 121 | 3 |
| 5 | Jin Yin-tan Hospital | 75 | 3 |
| 6 | The Central Hospital of Wuhan | 69 | 3 |
| 7 | Wuhan No.1 Hospital | 51 | 3 |
| 8 | Tongji hospital | 49 | 3 |
| 9 | Wuhan Pulmonary Hospital | 34 | 3 |
| 10 | Caidian District Maternal and Child Health Hospital | 26 | 3 |
| 11 | The Third People's Hospital of Hubei Province | 19 | 3 |
| 12 | Wuhan Asia Heart Hospital | 17 | 3 |
| 13 | Sixth Hospital in Wuhan | 13 | 3 |
| 14 | General Hospital of The Yangtze River Shipping | 12 | 3 |
| 15 | Wuhan Wuchang Hospital | 12 | 3 |
| 16 | Hubei General Hospital | 11 | 3 |
| 17 | Wuhan Hankou Hospital | 10 | 3 |
| 18 | Leishenshan Hospital | 8 | 3 |
| 19 | Puai Hospital | 6 | 3 |
| 20 | Fifth Hospital in Wuhan | 5 | 3 |
| 21 | Wuhan Third Hospital-Tongren Hospital of Wuhan University | 4 | 3 |
| 22 | Wuhan Hospital of Traditional Chinese Medicine | 4 | 3 |
| 23 | Wuhan Hanyang Hospital | 4 | 3 |
| 24 | CR Medical | 3 | 3 |
| 25 | Wuhan General Hospital of Guangzhou Military Area Command | 3 | 3 |
| 26 | Zhongnan Hospital of Wuhan University | 2 | 3 |
| 27 | China People's Liberation Army 457th Hospital | 2 | 3 |
| 28 | Wuhan Dongxihu District People's Hospital | 2 | 3 |
| 29 | China People's Liberation Army Central Theater General Hospital | 1 | 3 |
| 30 | Zaoyang First People's Hospital | 1 | 3 |
| 31 | CR medical | 1 | 3 |
| 32 | Hubei Province 672 Orthopaedic Hospital of Integrated Traditional Chinese and Western Medicine | 1 | 3 |
| 33 | Wuhan No.8 Hospital | 1 | 3 |
| 34 | Wuhan Red cross Hospital | 115 | 2 |
| 35 | Wuhan Commercial and Vocational Hospital | 4 | 2 |
| 36 | Wuhan No.7 Hospital | 3 | 2 |
| 37 | Wuhan No.9 Hospital | 3 | 2 |
| 38 | The Second People's Hospital of Dongxihu District, Wuhan | 3 | 2 |
| 39 | Wuhan No.10 Hospital | 2 | 2 |
| 40 | The First People's Hospital of Jiangxia District | 1 | 2 |
| 41 | Hubei Province Rongjun Hospital | 1 | 2 |
|  | NA | 343 |  |
| **No.** | **Fangcang shelter hospital** | **N** |  |
| 1 | Jianghan Fangcang Shelter Hospital | 277 |  |
| 2 | Jiangan Fangcang Shelter Hospital | 66 |  |
| 3 | Zhuankou Fangcang Shelter Hospital | 13 |  |
| 4 | Jianghan Economic Development Zone Fangcang Shelter Hospital | 12 |  |
| 5 | Hanyang Fangcang Shelter Hospital | 9 |  |
| 6 | Wuchang Fangcang Shelter Hospital | 8 |  |
| 7 | East Lake Rihai Fangcang Shelter Hospital | 6 |  |
| 8 | Provincial Party School Fangcang Shelter Hospital | 6 |  |
| 9 | Hanyang Sports School Fangcang Shelter Hospital | 4 |  |
| 10 | Dongxihu Fangcang Shelter Hospital | 2 |  |
| 11 | Wuhan Economic Development Zone Fangcang Shelter Hospital | 2 |  |
| 12 | Jiangxia Dahuashan Fangcang Shelter Hospital | 1 |  |
| 13 | Qiaokou Fangcang Shelter Hospital | 1 |  |
| 14 | Huangpi Fangcang Shelter Hospital | 1 |  |

‡ Hospitals in China are divided into 1(primary healthcare), 2, and 3 (High level of specialist healthcare) tier according to different functions and tasks.

# Table S3 The basic configuration of Fangcang shelter hospitals and a representative of the designated hospitals

|  | **Fangcang shelter hospitals** | **Jin Yin-tan hospital (a representative of designated hospital)** |
| --- | --- | --- |
| **Capacity (beds)** | 200-3000 beds (the net usable area of each bed is not less than 6㎡).  Take every 20 beds as a unit, and set up necessary partition facilities. There are 5-10 toilets for every 100 beds. | 900 beds |
| **The ratio of health workers to patients** | Medical stuff shall be assigned according to the bed-to-nurse ratio of 1:0.2 and the doctor-to-nurse ratio of 1:5. Medical staff implement a 24-hour shift system, with a shift every 6 hours. | Medical staff shall be assigned according to the bed-to-nurse ratio of 1:0.3 and doctor-to-nurse ratio of 1:3. Medical staff implement a 24-hour shift system, with a shift every 4 hours. |
| **Functional districts** | Ward, Intensive Care and Treatment Unit, Image Review Area, Clinical Inspection Area, Nucleic Acid Test Area | Department of Infectious Diseases, Department of Tuberculosis, Department of Hepatology, Department of AIDS, Department of Oncology, Department of Gastroenterology, Department of Traditional Chinese Medicine, Department of Integrative Medicine, Department of Thoracic Surgery, Department of Orthopaedic Surgery, Department of General Surgery, Department of Brain Surgery, Department of Trauma Surgery, Department of Emergency Medicine, Department of Critical Care Medicine, Anesthesiology, Interventional Ultrasound, Stomatology, Ophthalmology, Obstetrics and Gynecology and other clinical departments. There are Laboratory Medical, Medical Imaging Department, Pathology, Respiratory Intervention Department, Function Department, Pharmacy Department and other medical and technical departments. |
| **Medical devices and care provided** | Ward: It is an area where patients live and receive medical treatment and observation consisting of fixed wards and mobile wards. It provided commonly used drugs, infusion support, oxygen supplement, stethoscope, electronic sphygmomanometer, thermometers, SaO_2_ monitors, wheelchairs, and flat wagons. Intensive Care and Treatment Unit: It provided necessary rescue medicine, simple respirator, monitor, defibrillator, non-invasive ventilator, transport flat wagon, etc. Image Review Area: This area is composed of multiple groups of imaging vehicles, and provides various imaging inspection work such as X-ray, CT, and ultrasound. Clinical Inspection Area: This area is composed of multiple groups of inspection vehicles, that undertake blood routine examination and other laboratory inspection tasks. Nucleic Acid Test Area: It is composed of temporary P2 or mobile P3 laboratories, and undertakes the task of nucleic acid test for COVID-19. | A large specialized hospital capable of diagnosing and treating various infectious diseases such as liver disease, tuberculosis, lung disease, etc. There is a laminar flow operating room, the first negative pressure ward in Wuhan, and a P2 laboratory. Equipped with NMR, 64-slice CT, digital X-ray machine, ECMO, DSA system, thoracoscope, mediastinoscope, EUS, chromoscope ultrasonic diagnostic equipment, pulmonary function tester, artificial liver blood purification system, AEROSET, FCM, gene analyzer, PCR, real-time qPCR and other advanced medical equipment. |

# Table S4 Self-reported symptoms of COVID-19 patients and non-COVID-19 controls

|  | **Matched non-COVID-19 controls (n=1455)** | **Total COVID-19**  **(n=1455)** | | **Fangcang shelter hospital group (n=283)** | | **Designated hospital group**  **(n=1172)** | |
| --- | --- | --- | --- | --- | --- | --- | --- |
|  | **Prevalent symptoms**‡ | **Prevalent symptoms** | **Sequelae symptoms**† | **Prevalent symptoms** | **Sequelae symptoms** | **Prevalent symptoms** | **Sequelae symptoms** |
| **Any one of the following symptoms** | 353 (32.7%) | 1234 (85.1%) | 894 (61.4%) | 231 (81.6%) | 165 (58.3%) | 1003 (85.9%) | 729 (62.2%) |
| Fatigue or muscle weakness | 61 (5.6%) | 668 (46.1%) | 523 (35.9%) | 125 (44.2%) | 101 (35.7%) | 543 (46.3%) | 422 (36.0%) |
| Sleep difficulties | 141 (13.1%) | 552 (38.1%) | 303 (20.8%) | 100 (35.3%) | 52 (18.4%) | 452 (38.6%) | 251 (21.4%) |
| Hair loss | 93 (8.6%) | 541 (37.3%) | 215 (14.8%) | 98 (34.6%) | 34 (12%) | 443 (37.8%) | 181 (15.4%) |
| Smell disorder | 4 (0.4%) | 130 (9%) | 104 (7.1%) | 20 (7.1%) | 17 (6%) | 110 (9.4%) | 87 (7.4%) |
| Palpitations | 52 (4.8%) | 440 (30.3%) | 285 (19.6%) | 75 (26.5%) | 48 (17%) | 365 (31.1%) | 237 (20.2%) |
| Decreased appetite | 9 (0.8%) | 92 (6.3%) | 79 (5.4%) | 19 (6.7%) | 17 (6%) | 73 (6.2%) | 62 (5.3%) |
| Taste disorder | 3 (0.3%) | 107 (7.4%) | 105 (7.2%) | 20 (7.1%) | 20 (7.1%) | 87 (7.4%) | 85 (7.3%) |
| Dizziness | 63 (5.8%) | 369 (25.4%) | 217 (14.9%) | 61 (21.6%) | 30 (10.6%) | 308 (26.3%) | 187 (16.0%) |
| Nausea or vomiting | 5 (0.5%) | 55 (3.8%) | 40 (2.7%) | 12 (4.2%) | 8 (2.8%) | 43 (3.7%) | 32 (2.7%) |
| Chest pain | 19 (1.8%) | 250 (17.2%) | 205 (14.1%) | 41 (14.5%) | 37 (13.1%) | 209 (17.8%) | 168 (14.3%) |
| Sore throat or difficult to swallow | 8 (0.7%) | 134 (9.2%) | 74 (5.1%) | 26 (9.2%) | 13 (4.6%) | 108 (9.2%) | 61 (5.2%) |
| Skin rash | 4 (0.4%) | 186 (12.8%) | 111 (7.6%) | 30 (10.6%) | 16 (5.7%) | 156 (13.3%) | 95 (8.1%) |
| Myalgia | 10 (0.9%) | 243 (16.8%) | 193 (13.3%) | 51 (18%) | 39 (13.8%) | 192 (16.4%) | 154 (13.1%) |
| Headache | 32 (3%) | 212 (14.6%) | 115 (7.9%) | 48 (17%) | 28 (9.9%) | 164 (14%) | 87 (7.4%) |
| Cough | 27 (2.5%) | 165 (11.4%) | 100 (6.9%) | 26 (9.2%) | 15 (5.3%) | 139 (11.9%) | 85 (7.3%) |
| Joint pain | 99 (9.2%) | 577 (39.8%) | 298 (20.5%) | 108 (38.2%) | 62 (21.9%) | 469 (40%) | 236 (20.1%) |

Data are n (%) and mean (SD). ‡Prevalent symptoms are defined as the existing symptoms at follow-up. †Sequelae symptoms are defined as new and persistent symptoms, or symptoms that are worse than those before COVID-19 infection and that cannot be explained by other illnesses.

# Table S5 Detailed results of EQ-5D-5L questionnaire of the COVID-19 patients

|  | **Total**  **(n=1455)** | **Fangcang shelter hospital group**  **(n=283)** | **Designated hospital group**  **(n=1172)** |
| --- | --- | --- | --- |
| **Mobility** |  |  |  |
| No problems with walking around | 1350/1451 (93%) | 275 (97%) | 1075/1168 (92%) |
| Slight problems with walking around | 86/1451 (6%) | 6 (2%) | 80/1168 (7%) |
| Moderate problems with walking around | 6/1451 (0%) | 1 (0%) | 5/1168 (0%) |
| Severe problems with walking around | 5/1451 (0%) | 1 (0%) | 4/1168 (0%) |
| Unable to walk around | 4/1451 (0%) | 0 | 4/1168 (0%) |
| **Personal care** |  |  |  |
| No problems with washing or dishing | 1435/1451 (99%) | 283 (100%) | 1152/1168 (99%) |
| Slight problems with washing or dishing | 10/1451 (1%) | 0 | 10/1168 (1%) |
| Moderate problems with washing or dishing | 4/1451 (0%) | 0 | 4/1168 (0%) |
| Severe problems with washing or dishing | 0 | 0 | 0 |
| Unable to wash or dish | 2/1451 (0%) | 0 | 2/1168 (0%) |
| **Usual activities (e.g. work, study, housework, family or leisure activities)** | | |  |
| No problems with usual activities | 1417/1451 (98%) | 283 (100%) | 1134/1168 (97%) |
| Slight problems with usual activities | 25/1451 (2%) | 0 | 25/1168 (2%) |
| Moderate problems with usual activities | 4/1451 (0%) | 0 | 4/1168 (0%) |
| Severe problems with usual activities | 3/1451 (0%) | 0 | 3/1168 (0%) |
| Unable to do usual activities | 2/1451 (0%) | 0 | 2/1168 (0%) |
| **Pain/discomfort** |  |  |  |
| No pain/discomfort | 921/1451 (63%) | 191 (67%) | 730/1168 (63%) |
| Slight pain/discomfort | 417/1451 (29%) | 76 (27%) | 341/1168 (29%) |
| Moderate pain/discomfort | 98/1451 (7%) | 15 (5%) | 83/1168 (7%) |
| Severe pain/discomfort | 15/1451 (1%) | 1 (0%) | 14/1168 (1%) |
| Extreme pain/discomfort | 0 | 0 | 0 |
| **Anxiety/depression** |  |  |  |
| No anxiety/depression | 1046/1451 (72%) | 207 (73%) | 839/1168 (72%) |
| Slight anxiety/depression | 322/1451 (22%) | 68 (24%) | 254/1168 (22%) |
| Moderate anxiety/depression | 72/1451 (5%) | 6 (2%) | 66/1168 (6%) |
| Severe anxiety/depression | 11/1451 (1%) | 2 (1%) | 9/1168 (1%) |
| Extreme anxiety/depression | 0 | 0 | 0 |

Data are n (%) or n/N (%). The differing denominators used indicate missing data.

# Table S6 Extra-pulmonary organ performance of the community-based COVID-19 patients

| **Laboratory tests** | **Total**  **(n=1455)** | **Fangcang shelter hospital group**  **(n=283)** | **Designated hospital group**  **(n=1172)** |
| --- | --- | --- | --- |
| Leukocyte count, × 10^9^ per L | 5.8 (1.4) | 5.8 (1.5) | 5.8 (1.4) |
| <4 | 108/1434 (7.5%) | 25/276 (9.1%) | 83/1158 (7.2%) |
| Lymphocyte count, × 10^9^ per L | 1.9 (0.6) | 2.0 (0.6) | 1.9 (0.6) |
| <0.8 | 8/1434 (0.6%) | 1/276 (0.4%) | 7/1158 (0.6%) |
| Haemoglobin, g/dL | 134.6 (13.9) | 135.9 (14.4) | 134.6 (13.8) |
| Anemia | 52/1434 (3.6%) | 6/276 (2.2%) | 46/1158 (4.0%) |
| Platelet count, × 10^9^ per L | 197.4 (51.8) | 203.3 (51.5) | 196.0 (51.8) |
| <100 | 21/1434 (1.5%) | 0/276 (0) | 21/1158 (1.8%) |
| Albumin, g/L | 44.5 (2.2) | 44.6 (2.2) | 44.5 (2.2) |
| Alanine aminotransferase, U/L | 18.3 (17.8) | 18.7 (19.4) | 18.2 (17.4) |
| >40 | 93/1435 (6.5%) | 17/276 (6.2%) | 76/1159 (6.6%) |
| Aspartate aminotransferase, U/L | 23.3 (11.2) | 22.4 (9.1) | 23.5 (11.6) |
| >40 | 58/1435 (4.0%) | 10/276 (3.6%) | 48/1159 (4.1%) |
| Creatinine, μmol/L | 71.8 (29.2) | 68.8 (14.8) | 72.5 (31.6) |
| Creatinine > 133μmol/L | 13/1435 (0.9%) | 0/276 (0) | 13/1159 (1.1%) |
| eGFR, ml/min | 88.1 (26.8) | 96.1 (26.3) | 86.4 (26.6) |
| ≥90 | 608/1435 (42.4%) | 137/276 (49.6%) | 471/1159 (40.6%) |
| 60-89 | 662/1435 (46.1%) | 131/276 (47.5%) | 531/1159 (45.8%) |
| < 60 | 165/1435 (11.5%) | 8/276 (2.9%) | 157/1159 (13.5%) |
| HbA1C, % | 5.8 (1.1) | 5.7 (1.1) | 5.9 (1.1) |
| ≥6.5% | 196/1437 (13.6%) | 26/277 (9.4%) | 170/1160 (14.7%) |
| NT-proBNP, pg/ml | 162.9 (285.8) | 117.7 (92.0) | 173.7 (314.0) |
| Throat swab nucleic acid test positive | 0 | 0 | 0 |

Data are n (%) and mean (SD). Percentages are calculated by category after exclusion of missing data for that variable. P values comparing groups are from a χ² test for categorical variables, and Mann-Whitney U test for continuous variables. eGFR=estimated glomerular filtration rate. HbA1C= glycated hemoglobin A1C. NT-proBNP =NT-proB-type Natriuretic Peptide.

# Table S7 Post hoc analysis for sequelae symptoms by dividing patients with different follow-up duration

|  | **Overall**  **(N=1455)** | **<17 months group (N=378)¶** | **17 months group (N=893)¶** | **>17 months group (N=184)¶** |
| --- | --- | --- | --- | --- |
|  | OR (95% CI) | OR (95% CI) | OR (95% CI) | OR (95% CI) |
| **Age, years** | 1.00 (0.99-1.01) | 0.99 (0.97-1.01) | 1.00 (0.99-1.01) | 1.00 (0.97-1.03) |
| **Sex, women** | **1.48 (1.16-1.88)** | 1.07 (0.66-1.73) | **1.50 (1.11-2.04)** | **2.40 (1.21-4.77)** |
| **Cigarette smoking** | 0.80 (0.56-1.13) | 0.86 (0.45-1.66) | 0.81 (0.52-1.28) | 0.54 (0.16-1.81) |
| **Pre-existing comorbidities** | 1.26 (0.99-1.61) | 1.07 (0.66-1.74) | 1.32 (0.97-1.79) | 1.40 (0.67-2.92) |
| **Severe illness** | **1.46 (1.01-2.10)** | 2.35 (0.99-5.58) | 1.38 (0.86-2.21) | 1.08 (0.45-2.63) |
| **No. of initial symptoms** | **1.31 (1.23-1.40)** | **1.40 (1.22-1.60)** | **1.31 (1.21-1.42)** | 1.17 (0.98-1.39) |
| **Public health intervention, Fangcang shelter hospital** | 0.99 (0.75-1.32) | 1.26 (0.72-2.18) | 0.98 (0.68-1.41) | 0.68 (0.27-1.67) |
| **COVID-19 vaccination** § |  |  |  |  |
| **1 dose** | 0.92 (0.71-1.17) | 1.03 (0.65-1.63) | 0.83 (0.60-1.15) | 1.20 (0.51-2.78) |
| **2 doses** | 0.85 (0.63-1.15) | 0.77 (0.33-1.76) | 0.90 (0.62-1.30) | 0.54 (0.24-1.22) |

¶COVID-19 patients were divided into three subgroups based on the duration from symptom onset to follow-up: <17 months group, 17 months group, and >17 months group. § All recovers in this study received COVID-19 vaccines after their infection.
